# Co‐occurrence of *ATXN3* and *ATXN2 *repeat expansions in Chinese ataxia patients with slow saccades

**DOI:** 10.1002/mgg3.663

**Published:** 2019-03-28

**Authors:** Chao Wu, Qiong Cai, Huajing You, Xiangxue Zhou, Dingbang Chen, Guiling Mo, Xunhua Li

**Affiliations:** ^1^ Department of Neurology, National Key Clinical Department and Key Discipline of Neurology The First Affiliated Hospital, Sun Yat‐sen University Guangzhou China; ^2^ Department of Neurology The East Area of the First Affiliated Hospital, Sun Yat‐sen University Guangzhou Guangdong China; ^3^ Guangzhou KingMed Center for Clinical Laboratory Co. Ltd Guangzhou Guangdong China

**Keywords:** *ATXN2*, *ATXN3*, modifier, oculomotor, spinocerebellar ataxia

## Abstract

**Background:**

The presence of more than one polyQ‐related gene within a single individual is a rare incidence, which may provide the potential opportunity to study the combined effects of these spinocerebellar ataxia (SCA) genes.

**Methods:**

We retrospectively analyzed genetic data from 112 SCA3 probands and found Patient 1 harbored expanded *ATXN2 *allele (33 repeats) and intermediate *TBP *allele (41 repeats), and Patient 2 with intermediate *ATXN2 *allele (32 repeats). Detailed clinical and oculomotor performances were investigated. The age at onset and oculomotor parameters of both patients were compared with matched pure SCA3 groups controlling either disease severity or CAG repeats.

**Results:**

Most of the clinical phenotypes and oculomotor characteristics of these two patients were common to typical SCA3 patients. Compared to pure SCA3 groups controlling disease severity, mild reduced horizontal saccade velocity could be detected in both patients. However, mild expansions of the *ATXN2 *allele seemed to have no influence on the age at onset of Patient 1 but might have a mild impact on Patient 2.

**Conclusion:**

Our study provides supporting evidence that mild expansions of *ATXN2 *may have modifying effects on SCA3 phenotype. Larger control series and longitudinal data are warranted to confirm our results.

## 1 INTRODUCTION

Spinocerebellar ataxia (SCA) type 2 and 3 are caused by cytosine–adenine–guanine (CAG)n repeat expansions in *ATXN2 *and *ATXN3 *genes (OMIM 601517, 607047), which are the most common types of SCAs in China (Wang et al., 2011). It has been proven that CAG repeat length is inversely related to age at onset of gait ataxia (AAO). However, that only explains 50%–80% of the variability of AAO, suggesting the involvement of additional modifying factors. Recent studies from EUROSCA and another 802 Chinese Han SCA3 patients showed that SCA3 subjects with an intermediate *ATXN2 *allele (27–32 CAG repeats) had an earlier AAO than subjects with shorter *ATXN2 *alleles (Chen et al., 2016; Raposo, Ramos, Bettencourt, & Lima, 2015; Tezenas du Montcel et al., 2014).

The presence of more than one polyglutamine (polyQ)‐related gene repeat expansions within a single individual is a rare incidence. Combination of SCA3 and SCA17 in China, SCA2 and SCA10 in America, and SCA8 coexisting with SCA1, SCA3, or SCA6 had been reported previously (Kapur & Goldman, 2012; Xu et al., 2010). These patients could provide unique opportunity to study the potential genetic interactions between different SCA genes and their effects on clinical manifestation, which have been of major interests in recent degenerative cerebellar ataxia research.

Herein, we reported two patients who carried repeat expansions in both SCA2 and SCA3 genes, one of them also carried an intermediate *TBP *allele. Furthermore, we analyzed the combined effects of these polyQ disease genes on AAO and clinical phenotype including eye movement abnormalities at the individual level.

## 2 METHODS

### 2.1 Editorial policies and ethical considerations

Ethics approval was obtained by the ethics board of the Ethics Committee of the First Affiliated Hospital of Sun Yat‐sen University, Guangdong, China [No. (2014) 23]. The study was carried out only after written informed consent was obtained from all participants. Blood samples were also collected after obtaining written informed consent.

### 2.2 Participants and clinical information

Participants were recruited in the Neurogenetic outpatient clinic of the First Affiliated Hospital of Sun Yat‐Sen University, Guangdong, China, from October 2014 to April 2018. We retrospectively analyzed genetic data from 112 SCA3 probands and found two patients who harbored repeat expansions in both SCA2 and SCA3 genes. To measure the severity of ataxia, we used the Scale for the Assessment and Rating of Ataxia (SARA) that has been validated. Eye movements were recorded using video‐oculography following a central oculomotor battery that we described before (Wu et al., 2017). AAO was determined by a structured interview in which the patient and close family members were asked about the age of start of permanent and progressive gait instability.

AAO of both reported patients was compared with matched pure SCA3 groups controlling CAG repeats, namely AAO 1 (*n* = 12) and AAO 2 (*n* = 16) group, respectively. Oculomotor parameters of both patients were also compared with matched pure SCA3 groups controlling disease severity represented by SARA scores, namely Oculo 1 (*n* = 13) and Oculo 2 (*n* = 12) group, respectively. Pure SCA2 group (*n* = 6), one SCA17 patient, and normal controls (NC) (*n* = 26) were also recruited to compare the oculomotor characteristic between different genotypes (Table 1). For descriptive statistics of demographic, genetic, and measurable oculomotor parameters, means and standard deviations (*SD*) were obtained. Oculomotor data, which is out of the range of mean ± 2 *SD*, is defined to be abnormal.

**Table 1:** Clinical characteristics and oculomotor parameters of participants

Characteristics	Patient 1	SCA3 AAO 1	SCA3 Oculo 1	Patient 2	SCA3 AAO 2	SCA3 Oculo 2	**SCA2**	**SCA17**	**NC**
No.	1	12	13	1	16	12	6	1	26
Age at onset of gait ataxia, years	28	25.6 ± 5.5	NA	30	37.3 ± 5.8	NA	30.5 ± 6.4	33	NA
Disease duration, years	5	NA	3.7 ± 1.6	4	NA	5.3 ± 2.1	3.7 ± 2.3	7	NA
CAG repeat length	*ATXN2* (22,**33**)^a^ *ATXN3* (27,**77**) *TBP* (36,**41**)	77 ± 1.3	74.2 ± 3.7	*ATXN2 *(**32**, 22) *ATXN3 *(**74**, 23) TBP (36, 37)	74 ± 0.8	73.7 ± 2.1	43.2 ± 3.0	(**53**, 36)	NA
SARA score	6.5	NA	6.42 ± 1.1	12	NA	12.0 ± 1.4	10.1 ± 3.8	8	NA
Oculomotor signs									
Frequency of SWJ (Hz)	1.5	NA	0.75 ± 0.54	2	NA	0.83 ± 0.68	0	0.6	0.06 ± 0.13
Frequency of horizontal GEN (Hz)	1.5	NA	1.42 ± 0.40	1	NA	2.0 ± 0.88	0	0.6	0.09 ± 0.15
Horizontal peak saccade velocity (°/s)	301.2^b^	NA	552.9 ± 95.6	288.5^a^	NA	517.4 ± 103.3	156.8 ± 42.3	492.7	524 ± 56.9
Horizontal saccadic accuracy (%)	83.75	NA	94.8 ± 16.0	97.5	NA	88.8 ± 15.0	97.1 ± 24.7	92	98.2 ± 3.5
Upward peak saccade velocity (°/s)	208	NA	380.0 ± 128.9	NA^c^	NA	309.5 ± 70.7	238.0 ± 124.4	465	563 ± 100.5
Total antisaccadic error rate (%)	62.5	NA	62.2 ± 26.1	30.8	NA	73.7 ± 17.5	27.7 ± 29.6	100	19.2 ± 14.0

Data are given as mean ± *SD*.

^a^Values in bold represents longer repeats of the SCA gene.

^b^Represents data out of the range of mean ± 2 *SD*.

^c^Patient 2 had vertical gaze palsy.

Abbreviations: AAO: age at gait ataxia onset; GEN: gaze‐evoked nystagmus; NA: not available; NC: normal controls; SARA: Scale for the Assessment and Rating of Ataxia; SCA: spinocerebellar ataxia; SWJ: square‐wave jerk.

### 2.3 Genetic sequencing

CAG repeat size in the *ATXN3 *gene and seven other polyQ‐related genes (*ATXN1*, *ATXN2*, *CACNA1A*, *ATXN7*, *PPP2R2B*, *TBP*, and *ATN1*) was measured by polymerase chain reaction amplification of CAG tracts in combination with capillary electrophoresis in Guangzhou KingMed Center for Clinical Laboratory. Intermediate‐length CAG/CAA expansions in the SCA17 *TBP *gene (OMIM 600075) have been assumed to be 41–48 (Xu et al., 2010).

## 3 RESULTS

### 3.1 Clinical and genetic findings of patients co‐occurrence of *ATXN3* and *ATXN2*

**Figure 1:**
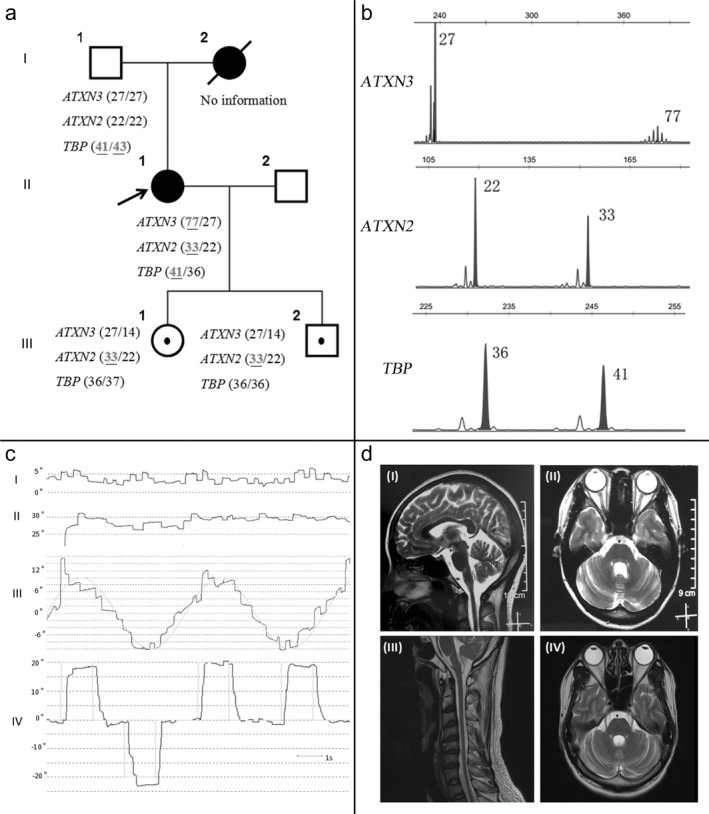
(a) Family pedigree of Patient 1. Squares indicate men; circles, women; diagonal lines, deceased. An arrow indicates the proband. Open shapes represent healthy members; black solid shapes represent symptomatic patients; open shapes with dark spot in the middle represent asymptomatic individuals with expanded repeats. The mutation statuses of the SCA2, SCA3, and SCA17 genes are shown next to the symbol of each person. (b) Genotyping results of *ATXN3*, *ATXN2*, and *TBP *of Patient 1. (c) Oculomotor deficits of Patient 1. I: Central fixation was severely impaired by square‐wave jerk. II: Nystagmus was found during horizontal gaze. III: Severe abnormal eye drift during smooth pursuit in horizontal directions. IV: Slow peak saccade velocity could be detected during horizontal saccade. Symbols: gray full line = eye movement track; 0°= central position; positive or negative number degree = right or left position. (d) The brain magnetic resonance imaging (MRI) T2‐weighted image of Patients 1 (I, II) and 2 (III, IV). The sagittal (I, III) sections reveal brainstem, cerebellum, and spinal cord atrophy. The axial images (II, IV) represent atrophy of the cerebellum and pons.

Patient 1 (II‐1, Figure 1a) is a 32‐year‐old, right‐handed female originating from south China. She had a 5‐year history of gradually progressive unsteady gait. She had no previous history of brain injury, excessive drinking, and smoking. The first neurological examination revealed slightly slur speech, gaze‐evoked nystagmus (GEN), mildly slowed saccades, and diplopia during upward gaze. Spasticity and hyperreflexia of the lower limbs and Babinski sign could be detected, but with normal strength and sensation. The gait was slightly wide based and she could not tandem walk. She always felt fatigue but still could manage basic activities of her daily living. At the time of her presentation to the clinic, a 1.5T MRI showed brain stem and cerebellar atrophy (Figure 1d: I, II). Her mini‐mental state examination (MMSE) score was intact. Two years later, she developed a wide‐based ataxic gait with multiple falls occasionally, dysarthria, upper and lower limb dysmetria, and dysdiadochokinesia. Several oculomotor alterations could be detected in Patient 1 as presented in Figure 1c. Square‐wave jerk could be induced during central fixation and horizontal gaze. Horizontal smooth pursuit was severely impaired. Interestingly, slow horizontal and vertical saccade, highly characteristic of SCA2, appeared in Patient 1. Videos for details of oculomotor deficits in Patient 1 could be found in the [Link], [Link], [Link], [Link].

Mutation analysis revealed expanded heterozygous CAG expansion in *ATXN3 *(77‐repeats), *ATXN2 *(33‐repeats), and intermediate‐range CAG/CAA expansion in *TBP *(41‐repeats) in Patient 1 (Figure 1a: II and b). Her mother was significant for ataxia with onset in her third decade and died at 42 years old. Her 57‐year‐old father (Figure 1a: I‐1) had two intermediate‐range CAG/CAA expansions in *TBP *(41, 43‐repeats) but with no sign of unsteady gait during the follow‐up. Her two children only inherited expansions in *ATXN2 *(33‐repeats) and are now asymptomatic (Figure 1a).

To further confirm the modifying effect of the *ATXN2 *gene, we searched our database and interestingly found Patient 2. He is a 34‐year‐old Chinese male whose genetic evaluation revealed expansions in *ATXN3 *(74‐repeats) and *ATXN2 *(32‐repeats). He presented with congenital ptosis of his left eyelid and progressive ataxia for 4 years. Neurological examination revealed ophthalmoplegia during upward gaze, horizontal nystagmus, distinct slowed saccades, areflexia of the limbs, dysmetria, gait disturbance, dysarthria, and dysphagia. Brain MRI showed cerebellar atrophy (Figure 1d: III, IV).

### 3.2 Modifying effects of PolyQ genes at the individual level

It has been proven that CAG repeat length is inversely related to AAO. In order to evaluate the combined effects of other polyQ disease genes on AAO in patients harboring more than one expansion, additional effect on AAO from *ATXN3 *expansions should be avoided. We recruited 12 genetically confirmed symptomatic SCA3 patients with medium range (22/22) of *ATXN2* as AAO 1 group. The mean CAG repeat length of *ATXN3 *was 77 ± 1.3, ranging from 76 to 79 in AAO 1 group, which was comparable to Patient 1 (Table 1). The mean AAO in AAO 1 group was 25.6 ± 5.5 years, and the AAO of Patient 1 (28‐year‐old) fell into this range. Similarly, we recruited 16 SCA3 patients matched for CAG repeat length (mean 74 ± 0.8, ranging from 73 to 75, Table 1) as AAO 2 group. The mean AAO in the AAO 2 group was 37.3 ± 5.8 years, while the AAO of Patient 2 (30‐year‐old) was out of this range.

To further assess the effects of these genes on oculomotor performance, the confounding role of the disease severity should be eliminated. Other pure SCA genotypes and NC should also be included in this comparison. We recruited 13 SCA3 patients matched for SARA score (mean 6.42 ± 1.1) as Oculo 1 group, 6 SCA2 patients (mean SARA score 10.1 ± 3.8), 1 genetically confirmed SCA17 patient, and 26 NC. Mean and standard deviation performance metrics of the oculomotor parameters of all the participants are given in Table 1. Compared to Oculo 1 group and NC, Patient 1 had significantly reduced horizontal peak saccade velocity. Other oculomotor parameters including frequency of square‐wave jerk and horizontal gaze‐evoked nystagmus, horizontal saccadic accuracy, and total antisaccadic error rate were comparative between Oculo 1 group and Patient 1. The horizontal saccade velocity of SCA2 group with higher SARA score was remarkably decreased than other groups. Similarly, we recruited 12 SCA3 patients matched for SARA score (mean 12.0 ± 1.4) as Oculo 2 group. Compared to Oculo 2 group and NC, Patient 2 had significantly reduced horizontal peak saccade velocity just like Patient 1. The horizontal saccade velocity of SCA2 group was still slower (Table 1). Our SCA17 patient had a normal horizontal saccade velocity and increased total antisaccadic error rate.

## 4 DISCUSSION

Of interest, we identified the unique co‐occurrence of expansions in three different SCAs: type 2, 3 and 17 in Patient 1, and co‐occurrence of SCA2 and 3 in Patient 2. Most of the clinical phenotypes were common to typical SCA3 patients. Notwithstanding, our study showed that a mild expansion of the *ATXN2 *allele might reduce the horizontal saccade velocity.

It really provides a dilemma in genetic counselling the clinical significance of an allele with 32 or 33 CAG repeats in the *ATXN2 *gene. The boundary between pathological and intermediate CAG expansion of polyQ diseases is often blurry. A few SCA2 cases with repeat lengths between 33 and 34 units have been reported before (Costanzi‐Porrini et al., 2000; Fernandez et al., 2000). Intermediate repeat lengths (29–31) in *ATXN2 *were lately associated with increased risk for MSA in Chinese individuals (Zhou et al., 2018). However, the penetrance of this intermediate length is still ambiguous. Thirty‐three CAG repeats are rare in SCA2 but do appear in French, Italian, and Cuban SCA2 cohorts (Figueroa et al., 2017; Tezenas du Montcel et al., 2014). Therefore, recent studies from EUROSCA and other replication cohorts tend to set the minimum pathological expansion of *ATXN2 *in 33 repeats (Chen et al., 2016; Raposo et al., 2015; Tezenas du Montcel et al., 2014).

Expanded *ATXN2 *repeats (length > 30 units) were also closely related to other late‐onset neurodegenerative diseases, such as progressive supranuclear palsy and amyotrophic lateral sclerosis (ALS) (Ross et al., 2011). Intriguingly, there was an overall increased risk of ALS for those carrying 29–33 CAG length of *ATXN2*, which indicated a genetic overlap between SCA2 and ALS (Sproviero et al., 2017). Although expanded repeat carriers were also identified in frontotemporal lobar degeneration, Alzheimer's and Parkinson's disease patients, these were not significantly more frequent than in controls (Ross et al., 2011; Wang et al., 2015). However, there was no evidence of dystonia, dementia, parkinsonism, and motor neuron deficits including weakness and fasciculation during clinical investigation in both patients.

Of note, previous studies also identified nearly 0.2% healthy control carriers with CAG repeat lengths >30. In fact, most of these controls were currently below the average onset age observed in patient cohorts (>38 years old) and might develop disease at an older age. Caution should be taken when attributing specific disease phenotype to these repeat lengths (Ross et al., 2011). Our reported two patients were in the same situation.

The previous study from EUROSCA and another Chinese SCA3 cohort showed that AAO was influenced by an intermediate *ATXN2 *allele (27–32). However, these effects were not replicated in other alternative cohorts and just showed a tendency for earlier estimated onset in Azorean SCA3 cohort. The lack of replication was interpreted as considering the rarity of *ATXN2 *intermediate alleles in the replication populations, different ethnic or geographic origins of the affected subjects, and differences based on population versus hospital samples (Tezenas du Montcel, 2015). In the present study, we found that a mild expansion of the *ATXN2 *allele seemed not to influence the AAO of Patient 1 but might have a mild impact on Patient 2.

Oculomotor characteristics are recognized as distinguishing features among SCAs. Slow saccades is a key feature of SCA2 and could be detected in SCA3 but usually in late stage in our cohort (Wu et al., 2017). We found a mild reduced horizontal saccade velocity in both Patients 1 and 2, which could be the effect of the CAG expansions in *ATXN2*, or just a coincidence of the phenotypic heterogeneity in SCA3 alternatively. Larger control series and longitudinal data are warranted to confirm our results.

SCA17 appears rare in China (Xu et al., 2010). Few symptomatic patients only having 41 and 42 repeats were reported so far (Nanda, Jackson, Schwankhaus, & Metzer, 2007). A previous study represented no interaction between *TBP *and *ATXN*2 or *ATXN*3 genes. Given that the father of Patient 1 who carries two intermediate‐range CAG/CAA expansions in *TBP *was still asymptomatic, we assumed *TBP *did not play a role in AAO or clinical phenotype of Patient 1 to date.

There is still lack of other solid evidence to prove that the SCA2 allele is also contributing to the phenotype of these two patients. More detailed imaging including three‐dimensional volumetry and diffusion tensor imaging with novel tractography methods could be beneficial to target SCA2 genotype‐specific patterns of structural progression (Adanyeguh et al., 2018; Reetz et al., 2013). Moreover, an autopsy could show evidence of expanded polyQ repeats, which remains the final chance to examine one patient segregating several types of SCAs (Kim, Park, & Jeon, 2018). Given the evidence available now, we would like to consider *ATXN2 *behaving as a genetic modifier instead of a causative gene. As the variability in AAO is not completely explained by the effects of the causative and modifier sister genes, other genetic or environmental factors must also play a role. The intact mechanism by which the repeated sequences cause disease remains unknown.

## 5 CONCLUSION

Our study provides supporting evidence that repeat expansion of *ATXN2 *may be a genetic modifier in SCA3. Finding more than one polyQ related gene expansions in a patient provides a dilemma in genetic counseling the clinical significance. We will continue to track these families in the future, which may help us further understand the pathogenic mechanism of the biological relationships among these SCA genes.

## CONFLICTS OF INTEREST

The authors report no conflicts of interest.
